# Insights into the Medical Evaluation of Ekbom Syndrome: An Overview

**DOI:** 10.3390/ijms25042151

**Published:** 2024-02-10

**Authors:** Florina Madalina Mindru, Andrei-Flavius Radu, Adrian Gheorghe Bumbu, Ada Radu, Simona Gabriela Bungau

**Affiliations:** 1 Doctoral School of Biomedical Sciences, University of Oradea, 410087 Oradea, Romania; florina.mindru@gmail.com (F.M.M.); adaroman96@gmail.com (A.R.); sbungau@uoradea.ro (S.G.B.); 2 Department of Preclinical Disciplines, Faculty of Medicine and Pharmacy, University of Oradea, 410073 Oradea, Romania; 3 Department of Psycho-Neuroscience and Recovery, Faculty of Medicine and Pharmacy, University of Oradea, 410073 Oradea, Romania; 4 Department of Pharmacy, Faculty of Medicine and Pharmacy, University of Oradea, 410028 Oradea, Romania

**Keywords:** Ekbom syndrome, delusional parasitosis, delusional infestation, psychiatric disorder, antipsychotics, dopaminergic pathway, dopamine transporter system

## Abstract

Ekbom syndrome, also known as delusional parasitosis (DP) or delusional infestation, is an uncommon psychiatric disorder distinguished by an enduring conviction of parasitic infestation, persisting notwithstanding the presence of medical evidence to the contrary. Primarily affecting middle-aged women, DP can manifest either as isolated psychological distress or as a component within a more intricate psychiatric framework, substantially influencing the quality of life for affected individuals. Its pathophysiological mechanism involves uncertain dopaminergic imbalances and dysfunction in the dopamine transporter system. Dermatologists often play a pivotal role in diagnosis, as patients first seek dermatological assessments of their signs and symptoms. However, DP frequently originates from underlying psychiatric disorders or medical variables, manifesting with neurological and infectious causative factors. The diagnostic complexity is attributed to patients’ resolute convictions, leading to delayed psychiatric intervention. First-line DP treatment involves antipsychotics, with newer agents demonstrating promising prospects, but the lack of standardized protocols poses a significant therapeutic challenge. In this narrative review, both a comprehensive approach to this uncommon pathology and an update on the state of knowledge in this medical subfield focused on optimizing the management of DP are provided. The complexity of DP underlying its uncommon nature and the incomplete understanding of its pathophysiology highlight the need for further research through multicenter studies and multidisciplinary teams to enhance therapeutic efficacy and safety.

## 1. Introduction

A psychiatric disorder is defined as a condition that occurs when there is a noticeable disruption in an individual’s mental functioning, emotional control, or actions that is significant enough to be considered clinically relevant [1]. There is a wide array of diverse mental diseases, such as post-traumatic stress disorder [2], depression and anxiety disorders [3], eating disorders [4], schizophrenia, and bipolar disorders [5]. Among these numerous pathologies of a psychiatric nature, Ekbom syndrome has been identified as an uncommon disorder [6].

Ekbom syndrome, alternatively termed delusional parasitosis (DP) or delusional infestation, represents an uncommon psychiatric disease that is defined by a fixed belief in infestation with pathogens despite the obvious evidence. This condition may manifest as either a solitary expression of psychological distress or as part of a more intricate psychiatric scenario, significantly impeding normal daily and social functioning [7,8].

It is considered a rare disease [9,10] and is most common in middle-aged women [11]. According to all of the research data, it can be classified into three main categories: primary delusional parasitosis, secondary (functional) DP (secondary to other psychiatric diseases), and organic forms of delusional infestation (mostly linked to drug abuse, such as with cocaine and amphetamines, and other organic deficiencies) [12]. Furthermore, the pathophysiological mechanism is still uncertain, but different theories support the cause of dopaminergic imbalances and striatal dopamine transporter (DAT) dysfunction [13].

Individuals afflicted by this syndrome often report tactile sensations on the skin and hallucinations of bugs, despite these manifestations being imperceptible to others. Concurrent psychological disorders are frequently present along with the condition, which proves resistant to logic or evidence. Even if it is a psychiatric pathology, dermatologists are most often the ones who have to perform the diagnostic process, as these patients seek dermatological services for their complaints [14,15].

DP typically arises as a consequence of an underlying psychiatric condition, including schizophrenia, bipolar disorder, depression, anxiety, obsessive-compulsive disorder, and anxiety disorder. Moreover, secondary delusions of parasitosis may be linked to various medical factors, such as deficiencies in B12 and folate, hyperthyroidism, diabetes, and neuropathy [16,17].

Neurological conditions, such as dementia, multiple sclerosis, encephalitis, meningitis, and complications following neurosurgery, can also contribute to these delusions. Additionally, infectious causes such as tuberculosis, leprosy, syphilis, and human immunodeficiency virus (HIV), along with substance abuse involving methamphetamine use, acute cocaine use, and alcohol withdrawal, are potential factors. Furthermore, side effects from medications such as ketoconazole, ciprofloxacin, topiramate, amantadine, phenelzine, and steroids are also implicated in the development of this syndrome [12].

DP is mentioned in the fifth edition of the Diagnostic and Statistical Manual of Mental Disorders (DSM 5) in the chapter related to delusional disorders, with fixed and false beliefs in infection with different pathogens, in cases where the psychiatric manifestations must persist for at least one month. In order to confirm a diagnosis of DP, it is mandatory for the patient to meet the following two criteria: conviction of being infested despite evidence to the contrary and abnormal cutaneous sensations attributed to this belief [18].

The history of the disease is quite wide, as it has been described in different forms in terms of terminology throughout time; it was first mentioned around 1937–1938, when Karl Axel Ekbom, a Swedish neurologist, described it as the pre-senile delusion of infestation (i.e., Praeseniler Dermatozoewahn). Later, in 1946, Willson and Miller referred to this disorder as delusional parasitosis. Closer to the current medical practice, Roland Freudenmann and Peter Lepping chose the term delusional infestation for this condition. Regardless of the name used in the various medical practices (in the medical literature, it can also appear as parasitophobia or oracarophobia), this psychiatric disease refers to individuals who have a strong belief that they are infested with parasites, even if there is no objective evidence of this matter [19].

In the context of addressing DP, the paramount emphasis in therapeutic intervention lies in the establishment of a robust collaboration between the clinician and the patient, fostering a constructive therapeutic relationship. Patients, dealing with frustration and mistrust, may become noncompliant or seek alternative opinions if their concerns are not attentively addressed. Practitioners should maintain an objective stance, acknowledging and validating patients’ symptoms while understanding their significant impact on their daily lives. When discussing symptoms during clinical encounters, it is often advisable to take a neutral position, recognizing the absence of observable organisms currently while acknowledging that the patient is indeed suffering [20,21,22].

The primary treatment for delusions of parasitosis involves carefully using antipsychotics at low doses to minimize side effects. If symptoms reoccur after stopping antipsychotic medication, restarting the treatment is recommended. Second-generation antipsychotics such as aripiprazole, risperidone, olanzapine, and quetiapine are the ones that are most often prescribed. The drugs should be taken at the lowest dose possible to avoid side effects such as extrapyramidal symptoms, QT prolongation, and metabolic problems. Newer antipsychotics such as paliperidone, brexpiprazole, and lurasidone have better safety profiles, but there is not much evidence to support their use in DP, and they may not be widely used because they are expensive [23,24,25,26]. Ekbom syndrome is a medical challenge for clinicians because most of these patients are not compliant, apply self-medication methods, and arrive very late in psychiatric services.

The aim of the present study was to update the state of knowledge on this uncommon disorder, address and centralize the current information on medical practices and multiple approaches to this syndrome, and provide an overview of the overall management in a context where information needs to be continuously updated due to the gaps encountered, especially at the level of the complete elucidation of the pathophysiological mechanism for a more optimal treatment. The contribution to the scientific literature is based on the distinct and comprehensive organization of the most recent diagnostic and therapeutic approaches, as well as the presentation of the associated pathologies in detail.

## 2. Methodology of Research

The present manuscript addresses and filters scientific publications on the management of DP, highlighting the most relevant aspects (epidemiological, diagnostic, clinical, and therapeutic). To this end, a detailed search was performed in some of the largest databases covering medical topics (e.g., PubMed, SpringerLink, ScienceDirect, Nature, and Web of Science) while utilizing the Boolean operators AND and OR. The search algorithm was based on correlating the structure of the manuscript in terms of the most important elements underlying the optimal management of a disorder with logical operators that allowed the filtering of the results and the reduction of the search bias as much as possible.

The database search algorithm included the following search terms: “Ekbom syndrome”; “delusional parasitosis”; “Ekbom delusion”; “delusional infestation”; “management of delusional parasitosis”; “management of Ekbom delusion”; “management of delusional infestation”; “delusional parasitosis AND Ekbom syndrome AND delusional infestation”; “(treatment AND (delusional parasitosis OR Ekbom delusion OR delusional infestation))”; “(diagnosis AND (delusional parasitosis OR Ekbom delusion OR delusional infestation))”; “case reports AND delusional parasitosis”; “case reports AND Ekbom delusion”; “case reports AND delusional infestation”; “epidemiology AND delusional parasitosis”; “epidemiology AND Ekbom delusion”; “epidemiology AND delusional infestation”.

Publications that were not in English or that lacked relevant content for the present study and those that deviated from the article or book-type format were excluded. A precise selection process resulted in the citation of 141 bibliographic references, published from 1983 to 2023, to validate the data in this study.

## 3. Epidemiology and Pathophysiological Insights of DP

DP represents a relatively infrequent phenomenon that is seldom encountered by psychiatrists and dermatologists. This rarity is attributed to patients’ conviction that their symptoms are not delusional but grounded in reality, further leading to limited clinical visibility. The pathophysiological mechanisms underpinning delusions of parasitosis remain inadequately elucidated, posing a substantial challenge to comprehensive understanding within the scientific community [12].

### 3.1. Incidence and Prevalence

In many cases, DP can be classified as a shared psychotic disorder because approximately 5 to 15% of these patients share their symptoms with people from their family or their close social circles [27].

Prevalence and incidence data may vary depending on the region, but also with different periods of time. For the American continent, the initial studies concluded that the rate of delusional infestation is 1–1.9 per 100,000 person-years, and the average age at diagnosis is 61. Previous research suggested that DP affects women more frequently than men, although in younger populations, the ratio is thought to equal 1:1 [28,29].

If only the American population is considered, a prevalence of 3.7 per 100,000 was discovered in central California according to a groundbreaking study funded by the Centers for Disease Control and Prevention. The same study also reported that the average age was 52, and more than 75% of the patients were females [11].

As for the European continent, different data have been discussed. British researchers suggested annual incidence rates of 2–2.37 and 17 per 1 million inhabitants per year [30], and according to other studies, the prevalence is considered to be around 80 cases per million [31].

In the Indian population, previous studies have reported a prevalence of 19 out of 4200 patients, with an increase in incidence being noted after 40 years old. Women in this demographic appear to be at a higher risk than men [32].

By analyzing recent data from the literature, three recently published studies concluded that the general incidence of this disease ranges between 1.9 and 3.7 cases/100,000 subject-years [33,34] (with the most often reported being the first value of the incidence—namely, 1.9 cases/100.000 subject-years), and the prevalence was estimated at 27.3/100,000 subject-years [35]. DP can also occur, secondary to substance abuse, in teenagers and young adults [36].

### 3.2. Pathophysiological Approaches

Recent studies suggested the hypothesis of dopaminergic system imbalances as a possible etiopathogenic mechanism. It is important to draw a distinction between primary and secondary causes because in secondary DP, the psychiatric symptoms can occur incidentally with other primary psychiatric illnesses, such as schizophrenia spectrum disorder, organic brain diseases, vitamin B12 deficiency, and drug misuse [13].

One main theory for the pathophysiological mechanism involved in DP refers to the dopaminergic neurotransmission etiology, which is also common for both psychotic and delusional disorders. It is important to mention that DP secondary to the misuse of drugs, such as cocaine, amphetamine, or methylphenidate, can be explained by increased synaptic dopamine levels, which are caused by a blockage of presynaptic dopamine reuptake at the dopamine transporter [37]. Starting from these hypotheses, another widely acknowledged theory that can also support the etiopathogenesis of the disease involves the decrease in or blockage of the striatal dopamine transporter (DAT). The DAT is a presynaptic plasma membrane protein, and it is responsible for maintaining an appropriate level of dopamine in the intersynaptic space [38]. Its functions are altered with age due to the decrease in estrogen levels (as estrogen plays an important neuroprotective role). DAT dysfunction leads to higher extracellular striatal dopamine levels in the synapse, and as a consequence, this will determine disturbances in perception and thinking (tactile hallucinations and delusions) [14].

As a general overview of the pathophysiology in DP, studies proposed that the following hypotheses can explain the increased level of dopamine: (i) a DAT inhibitor factor that blocks the reuptake of dopamine; (ii) a decrease in DAT activity; (iii) dopamine-catabolizing enzyme disturbances; (iv) an age-related decline in DAT density [38,39].

Presynaptic auto-receptors, which can be present on the presynaptic neuron, act as feedback sensors for monitoring the level of released dopamine. Dopaminergic receptors can be classified into two main types: (i) D1-like receptors (which include D1 receptors with roles in cognition, motivation, and rewards, and D5 receptors, which have roles in learning and memory); (ii) D2-like receptors (which include D2 receptors associated with motor control, emotion, and cognitive function, as well as D3- and D4-like receptors, which have roles in emotional regulation, cognitive processes, and motor function) [40].

Taking all of these into account, as a main-line treatment, antipsychotics have the role of improving the altered dopamine transmission. Through medication, a blockage of the dopamine D2/D3 receptor in the ventral striatal regions can diminish the symptomatology [37,38].

**Figure 1:**
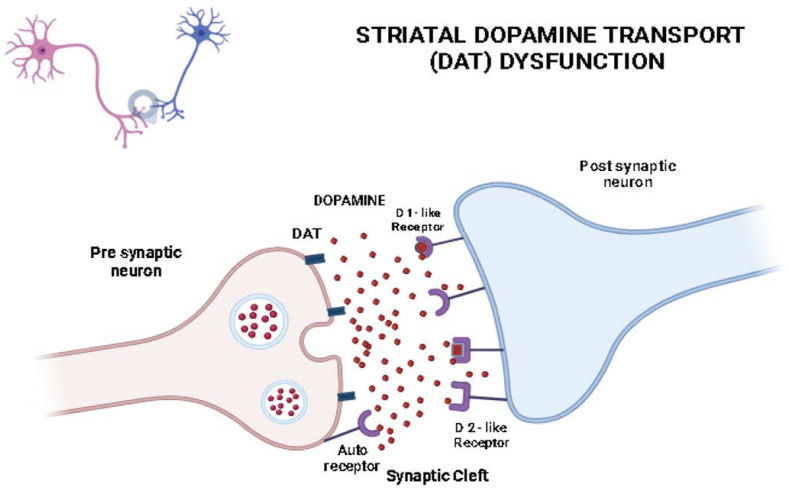
The involvement of the DAT in the pathogenesis of DP. Within this synapse, there are pre-synaptic terminals originating from dopaminergic neurons and post-synaptic receptors on the receiving neuron. The dopaminergic neuron synthesizes dopamine and stores it in vesicles in a healthy state. When an action potential reaches the pre-synaptic terminal, it triggers the release of dopamine into the synaptic cleft through exocytosis. After release, dopamine molecules bind to post-synaptic receptors, inducing the desired neurochemical effects. To regulate the duration of the signal, the dopamine, which is not bound to receptors, is swiftly cleared from the synaptic cleft by the DAT, which is present on the pre-synaptic membrane. In cases of dysfunction, the DAT fails to efficiently reuptake dopamine, leading to an accumulation of dopamine in the synaptic cleft and a prolonged presence of the neurotransmitter in the synaptic cleft. This persistent presence can result in overstimulation of post-synaptic receptors and contribute to psychiatric symptoms associated with altered dopamine signaling.

The mechanism involving the DAT system is depicted in Figure 1.

### 3.3. Risk Factors and Contributing Conditions

The exact cause of this rare disease has not been determined, but certain risk factors come into consideration, depending on the type of DP. Three categories have been described: primary DP, secondary or functional DP (which is usually associated with other psychiatric diseases, such as schizophrenia, anxiety, neurocognitive disorders, and depression), and organic forms of delusional infestation (mostly linked to vitamin B12 deficiency, infections, hypothyroidism, diabetes, postherpetic neuralgia, and substance abuse, especially cocaine, opiates, and methamphetamine) [11,12]. Another specific form is represented by ocular DP, which is a rare form, and patients mainly complain about parasites located in the eyes. They will use different methods to get rid of the supposed parasites and can injure themselves (periocular excoriations, papillary conjunctivitis, chemosis, conjunctival laceration and chemosis, corneal epithelial toxicity, and stromal ulceration) [41].

For methamphetamine users who subsequently develop DP, a particular syndrome called “meth mites” has been described. These patients describe the parasites as crawling under their skin [11].

Some of the most common risk factors, which are also considered the general ones, are related to socioeconomic problems, marriage status (e.g., divorce), a very stressful lifestyle, female gender, and old age [42]. Signs of cortical atrophy and vascular encephalopathy in elderly people can be linked to the risk factors in this case, independently of neurocognitive disorders [11]. Many studies concluded that social isolation was a general risk factor. More research might be performed after the lockdown for the COVID-19 pandemic to see how much the pandemic restrictions may have influenced vulnerable patients [37,43,44].

If the risk factors are approached strictly according to age group, the age limit of 40 is relevant. Thus, for those under this specific age, the main risk factor is drug abuse, and age groups over this limit include female gender, stress, loneliness, divorce, financial problems, and a disorganized lifestyle [11].

For patients with DP, it is also very important to consider any medication that was taken prior to the onset of symptoms. Studies have shown that some drugs are specifically related to DPs. For this case, the following drugs are included: corticosteroids, opiates, benzodiazepines, ketoconazole, fluoroquinolones, topiramate, pramipexole, and ropinirole [45].

One main feature that can trigger specific DP symptoms is the access of some patients to veterinary solutions, which can cause pruritus and other psychiatric signs and symptoms. One case report mentioned doramectin, an antiparasitic medication used only in animals such as cattle or sheep. Its toxicity has the ability to kinetically overload the transporter, resulting in greater central nervous system distribution, which leads to system damage [46].

## 4. Clinical Aspects of DP

The classic profile of a patient suffering from DP is represented by elderly women, who may or may not have associations with other psychiatric pathologies. As far as premorbid personality is concerned, studies have indicated that paranoid and obsessive-compulsive traits are noticeable in these patients [36,47].

The main symptoms of DP include delusions (the intense belief that the patient is infested with parasites) and tactile hallucinations (unusual cutaneous sensations such as “biting” or “crawling” on the skin or other parts of the body). Often, patients describes all of their problems in a very precise and detailed manner. Moreover, in an attempt to convince others, especially clinicians, of their suffering, patients will bring supposed evidence of infestation. This is considered a pathognomonic sign named the “matchbox sign”, “specimen sign”, or “pill-bottle sign” [36,48]. These samples brought for investigation include mostly skin flakes, mucus, hair, nails, synthetic or textile fibers, plant materials, insects, or nonpathogenic worms. Sometimes, the morphology of the specimens can be include natural patterns, which might lead to misinterpretation by the patient or even the clinician. Expert laboratory testing of samples from individuals suspected of having DP is very important to rule out an actual parasitic infection [49].

For an effective approach to patients, physicians must differentiate between primary and secondary forms. In primary DP, symptoms occur unexpectedly in comparison with secondary DP, which is related to other medical problems, of which the most significant would be not only other psychiatric conditions, such as schizophrenia, dementia, depression, and amphetamine or cocaine intoxication, but also somatic problems, such as diabetes, neuropathies, and cardiovascular disorders [47,50].

Although we are considering two forms of DP, the symptoms are similar. An important aspect that differentiates primary DP from secondary DP is the sequence of appearance of symptoms; the sense of tactile hallucinations is a significantly smaller part of the primary delusional infestation, where delusions come before the abnormal sensations. According to one proposed hypothesis, persons who have secondary delusional infestation initially have unusual perceptions that are then incorrectly assessed [48].

In a significant percentage of these cases, shared psychosis can occur. Thus, there can be discussions of folie a deux (two individuals), folie à trois (three persons), folie à plusieurs, folie à famille (more people or an entire family), or even DP by proxy, where the patient has a strong belief that others (even their pets) suffer from infestation (but these cases are less common in medical practice). Also, considering the impact of social media and the internet nowadays, the term “folie à internet” has also come to the attention of scientists [36,48].

This pathology can be considered a psychiatric emergency because risky behaviors may develop, such as setting fire to belongings, self-harm by using toxic substances in order to kill the worms, and even suicide attempts [36]. Another common symptom mentioned is the discomfort that the patients feel in their own houses, which leads to repetitive house-moving actions. Furthermore, relocations do not fix the problem, since those impacted by DP transfer their concerns from one setting to another in many instances [51].

DP can have an episodic or chronic course, and the duration may extend from days to years, with a 3-year average. Taking the etiology into account, primary DP has a chronic course compared to the second form, which can be brief if it is substance-induced. More than half of the patients with DP have a history of depression; therefore, both diseases may occur simultaneously. There are individuals for whom DP appears to cause depression and patients for whom depression appears to precipitate DP [52]. A very small number of patients with DP can recover without therapy [53].

## 5. Diagnosis of DP

The diagnostic process can sometimes be quite challenging, as these patients reach psychiatric services later in their evolution. Very common for them is self-treatment, which can cause nonmedication-related injuries, and usually, they go to dermatologists or infectious disease specialists for further investigations before being referred to a psychiatry department [54]. In the United Kingdom, there is a subspeciality called psychodermatology, which mainly deals with cases such as DP [55].

DP is categorized in the Delusional Disorder section and is of the somatic subtype according to the DSM 5 [18]. Delusional disorders are described as a persistent beliefs that re contrary to reality but are not accompanied by other prominent psychotic symptoms. The somatic subtype specifically involves delusions about bodily functions or sensations [8,36,48]. In the tenth revision of the International Statistical Classification of Diseases and Related Health Problems, DP is typically classified into the broader category of nonorganic psychotic disorders (the specific code for diagnosis is F22.8) [56].

Regarding diagnostic evaluation, the main symptoms include a false impression of being infested with pathogens without any medical evidence (this idea can rise to the level of delusion) and the presence of aberrant, typically qualitative, cutaneous sensations (in line with the ideas of infestation). Additional symptoms include hallucinations (visual and tactile); there is no particular part of the body that may be more affected [36].

Currently, there are no standard mental state examinations to facilitate the diagnostic process and determine the diagnosis and differential diagnosis. For the clinical interview, psychiatrists should consider sensorium and cognition, mood and affect, perception and thought content, insight, and risk assessment [57].

### 5.1. Paraclinical Diagnosis

For a proper evaluation, it is mandatory that all of the necessary tests be performed before the diagnosis of DP is confirmed in order not to overlook other possible underlying diseases. Laboratory tests are carried out first, and they include a full blood count and analyses of the erythrocyte sedimentation rate, C-reactive protein, liver function, thyroid-stimulating hormone, serum creatinine and electrolytes, fasting glucose, urine drug tests, etc. [29,58]. Also, serology for borrelia, treponema, hepatitis, and HIV infection, vasculitis screening, skin allergy tests, vitamin B12 levels, and folate levels must be considered [29,45,58]. Table 1 presents the main laboratory tests utilized for screening underlying organic conditions in patients diagnosed with DP [59,60,61,62,63,64,65,66].

Furthermore, if a patient presents with various lesions of the skin, a skin biopsy may be performed, with the patient choosing the collection site. In many cases, the most common findings are dermatitis or excoriations [45]. However, this approach should be done with caution in order not to encourage patients to ask for such investigations as a repetitive routine check-up [45,67].

In some medical contexts, patients may present to dermatologists after they have self-diagnosed themselves using certain devices, such as a dermatoscope. Often, these cases become a real challenge for dermatologists because, although further investigations rule out a possible infestation, patients are convinced by their evidence and absolutely refuse psychiatric examinations [68].

After all possible underlying organic conditions have been excluded, psychiatrists should consider other mental health problems, and for this reason, a screening for anxiety, depression, and suicidal behavior must be performed. Also, symptoms of dementia, schizophrenia, and delirium must be properly evaluated [29,58].

The available evidence does not definitively establish that distinct alterations in computed tomography or magnetic resonance imaging are exclusive to individuals with DP. However, a study of 18 DP patients in total compared to 20 healthy controls identified common patterns regarding cortical changes in the case group. Notably, DP patients exhibited wider right medial orbito-frontal gyrus cortical thickness, reduced surface area and folding in temporoparietal regions, and decreased surface area in various brain regions linked to psychotic disorders. These changes were spatially limited, primarily affecting psychosis-vulnerable areas, rather than being uniformly distributed across the entire brain [69].

Modern neuroimaging techniques could help with the understanding of the pathophysiology of DP, as well as that of how the brain correlates with antipsychotic therapy, but there are still very few data regarding these matters due to the restricted research in this domain [70].

### 5.2. Differential Diagnosis

The process of differential diagnosis is very important because the main symptoms in DP may be part of another possible underlying organic pathology, and for this reason, a multidisciplinary approach is an option to start with in most cases.

Formication is the first alternative diagnosis to be considered. In these contexts, patients experience various sensations, such as stinging or crawling, but they do not develop delusions. Moreover, formication may be linked to an underlying neurological condition such as multiple sclerosis or occur without an apparent cause and can also indirectly occur in menopause, exposure to pesticides or other chemicals, mercury poisoning, diabetic neuropathy, skin cancer, syphilis, Lyme disease, herpes zoster, and alcohol withdrawal [71,72].

A specific situation that can cause DP-like symptoms is that of cocaine users. Cocaine formication, which may appear, is also known as cocaine bugs. In these situations, detoxification programs are used as the first treatment strategy. Ideas of contamination and visual or tactile hallucinations may also develop in the context of a pre-existing psychiatric illness. In this case, schizophrenia and depression with psychotic elements are considered, and the treatment is chosen according to the underlying illness [71].

A genuine parasitosis can include scabies, pet-induced dermatitis, Grover’s disease, and environmental mites. In these situations, a biopsy and proper investigation can be required [8,71].

## 6. Therapeutic Management

Although the diagnostic criteria are quite explicit for DP, therapeutic assessment can be problematic [73].

Most studies to this point, as well as the conclusions of various case reports, recommend antipsychotics as the first line of treatment. The main pathophysiological mechanism on the basis of which treatment approaches have been developed is the dysfunction of the dopamine transporter (the amount of dopamine in the synaptic cleft rises when the DAT is not working properly, and this may explain the itching sensations that these patients feel) [74]. Elevated dopamine levels at the synapse appear to be associated with itching due to the activation of dopaminergic neurons in the ventral tegmental area and their projections to the nucleus accumbens. Studies suggest that scratching behaviors induced by itching stimulate these dopaminergic neurons, leading to increased dopamine release in the nucleus accumbens [75,76].

The first antipsychotic medication that was widely used to treat people with DP was pimozide. As other atypical antipsychotics have become available as therapeutic choices with fewer side effects, drugs such as olanzapine, risperidone, paliperidone, aripiprazole, and quetiapine were considered [77].

Some cases have registered resistance to treatment with olanzapine in doses of 5 mg/day and risperidone in doses up to 6 mg/day, although complementary methods of treatment (hypnotism) have also been associated. This shows that DP treatment can often be a challenge for psychiatrists [73].

Another case report demonstrated an experience with aripiprazole, which is a well-tolerated medication with a pharmacological profile that is distinct from those of other atypical antipsychotics. Due to its partial 5 hydroxy tryptamine 1A agonism, it has been suggested that it may decrease the feelings of sadness and anxiety that are often seen in individuals with DP. As a result, aripiprazole may be the treatment of choice for those who have DP, particularly if they are old, have cardiac issues, are depressed, or are anxious [78].

Studies related to quetiapine treatment are still unclear. In some cases, it has proved to be effective in reducing symptoms, but other patients have not responded to treatment. Doses have ranged from 25 to 800 mg/day [79].

Starting with a low dose and progressively increasing it every four weeks, depending on the response, is the general approach to therapy. During the treatment, patients should be evaluated and questioned about any possible side effects. The medication can be prescribed after an improvement is obtained for another 3 to 6 months. For other underlying psychiatric conditions, such as anxiety or depression, benzodiazepines and antidepressants can be associated with the treatment [80].

As for the treatment with pimozide, limited double-blinded crossover studies reported the effectiveness of pimozide at a dose of 2–7 mg/day. The main improvements during the pimozide treatment included itch alleviation and a decrease in delusions [81].

A few case studies included paliperidone depot as a treatment option for primary and secondary DP, but there are still not enough data to support its effectiveness. It can be considered a treatment option for patients with low compliance and no insight [82].

Due to the small number of cases, it is not possible to formulate a general hypothesis for the use of depot forms of antipsychotics in DP. Small studies on relatively low-dose fluphenazine decanoate have demonstrated efficacy in terms of remission probability [83].

Another case report with good results regarding remission and compliance included treatment with olanzapine pamoate depot. Furthermore, this is a treatment proposal, as there are no other cases cited in the literature, and clinicians should also consider the main side effects of this treatment—specifically, metabolic syndrome [84].

Psychiatrists are reserved concerning the use of long-acting injectable forms in DP. For senior individuals who have no prior history of psychotic symptoms, this therapeutic option is not advised because of the high risk of developing neuroleptic malignant syndrome [85].

Given that there are cases in the literature that did not respond to antipsychotic treatment, clinicians have been encouraged to expand their treatment strategies. DP is often associated with other psychiatric pathologies. A case study conducted by the Department of Psychiatry at Massachusetts General Hospital in Boston, Massachusetts, recommended evaluating DP patients for obsessive-compulsive disorder and considering selective serotonin reuptake inhibitor therapy, which is a first-line therapy in obsessive-compulsive disorder nonetheless [86]. Another case study in which the patient did not respond to first-line antipsychotics provided another treatment strategy with symptom reduction by combining electroconvulsive therapy with clozapine. Unfortunately, there are not many other examples proving the efficacy of electroconvulsive therapy, and for this reason, it remains an option for cases that are resistant to classical therapy plans [87].

Several additional investigations also proposed that combining an atypical antipsychotic with a selective serotonin reuptake inhibitor, such as fluoxetine, could be more effective. This drug association was particularly analyzed in animal studies; atypical antipsychotic drugs such as risperidone block dopamine receptors in the brain, among other neurotransmitter systems, and fluoxetine primarily increases the levels of serotonin in the synaptic cleft by inhibiting its reuptake. By combining these two types of medication, a synergistic effect can be achieved, and it is thought to enhance the release of dopamine in prefrontal areas more effectively than using either drug alone. Some potential advantages of this association include the following: improvements in cognitive performance, which can be particularly relevant for individuals with conditions characterized by cognitive deficits, such as delusional disorders; it can enhance treatment tolerance; it is possible to reduce the risk of side effects; it is possible to achieve therapeutic effects with lower doses of each drug [88,89].

So far, no standard of treatment has been established. Comparative studies have shown that both typical and atypical antipsychotics have similar results. In general, atypical antipsychotics are more widely used due to their fewer side effects. A comprehensive recommendation for healthcare practitioners would involve a meticulous consideration of individual patients’ characteristics, encompassing pertinent medical comorbidities, alongside a discerning evaluation of the respective side effect profiles and safety parameters inherent to each antipsychotic agent. Such a judicious approach to the selection of antipsychotics is imperative in order to optimize therapeutic outcomes while minimizing potential adverse effects [90,91]. Table 2 provides a brief overview of antipsychotics that can be used for the therapeutic management of DP.

All general treatment guidelines recommend antipsychotics for DP, but there are many patients who have no improvements. Studies are limited in terms of the effectiveness of combining psychological treatments. In some cases, by applying psychotherapeutic methods, an improvement can be observed in terms of emotional distress associated with hallucinations, but insight into the disease is still absent. One case report mentioned cognitive therapy behavior as complementary to the antipsychotic treatment, but a different response secondary to this treatment strategy could not be concluded upon as a final result. Despite the fact that the use of a psychological therapeutic model is considered a possibility for delusional infestation, it is not possible to draw the conclusion that this form of psychotherapy is a successful overall treatment for this illness [97].

Without therapy, this illness may last decades, with symptom durations of even up to 30 years [98]. It is well known that early interventions are advisable in early psychotic episodes, but there are no data to sustain how this principle can be applied for patients with DP, as they arrive late in psychiatric services. Nevertheless, early intervention initiatives for DP patients should be delivered in collaboration with mental health doctors and other clinicians as much as possible [99].

Another aspect that should be mentioned is the self-medication that patients apply, especially in relation to antiparasitic therapy. Antiparasitic treatment is not required because it maintains and encourages the patient’s delusions. Antiparasitic agents, while employed for therapeutic purposes, are not devoid of potential adverse effects, which encompass neurotoxic manifestations and allergic reactions. Patients may exhibit a proclivity to misuse these agents, persistently seeking their prescriptions even in the absence of substantiated efficacy. This context places healthcare practitioners in a recurrent dilemma, entailing the administration of a medication devoid of evident clinical indication. The prescription of irrelevant medicine may indicate a physician’s misdiagnosis. It might also imply compliance with patient requests rather than referral for a proper mental evaluation [100].

### The Importance of a Multidisciplinary Approach

The entire process of treatment for patients with DP can sometimes be problematic, and this can be one of the reasons for why it is very important for physicians to be able to build an effective doctor–patient relationship for the maintenance of the compliance of the patients. Therefore, for proper management, a multidisciplinary approach is recommended, especially because these patients are considered to be a medical challenge most of the time [101,102,103].

DP is seen more often in older patients, and the risk of their presenting other medical comorbidities is quite high; therefore, a diagnosis can sometimes be difficult to establish. Proper collaboration among different specialists is important for these patients to improve their clinical outcomes [103].

A dermatological examination must always be performed because DP can lead to different self-induced cutaneous lesions, which require additional treatment most of the time. Also, collaboration between dermatologists and psychiatrists is important for the treatment of other possible side effects of different antipsychotics, which are the first-line treatment for this pathology [103,104].

When addressing DP within the gastrointestinal tract, patients should also be evaluated by gastroenterologists because the interplay of gastrointestinal symptoms and psychiatric disorders is already widely known. Moreover, antipsychotics can cause not only gastrointestinal symptoms but also disturbances in the levels of cholesterol, an increase in the risk of metabolic syndrome, and arrhythmias, among other possible side effects. This is another strong reason for a multidisciplinary approach to these patients, so cardiologists and internists can also be involved [103,104].

Collaboration with pharmacologists can also be helpful in monitoring antipsychotic levels to detect any possible poor response to the therapy and prevent any drug interactions [104].

Compliance with therapy by patients with mental diseases is inconsistent and difficult. Factors that contribute to this matter are an age over 60, a stressful workload, a negative attitude toward medicine, a lack of insight, and a feeling of stigmatization by family and health professionals. Some of these factors can also be seen in patients with DP. Psychiatrists can take psychological interventions into consideration as an extended strategy of treatment. With different forms of therapy, patients can develop a better understanding of the disease, improve their social adaptability, learn how to trust their physician, and build better compliance with the treatment [97,101,105].

## 7. DP and Associated Diseases

### 7.1. DP and Folie à Deux

The incidence of folie à deux encountered in DP is about 5–15%. Usually, only one patient (the primary case) has the psychotic disorder, and that patient causes symptoms in the other one (who is a member of the family). Also, there are four categories for folie à deux: folie imposée, folie simultané, folie communiquée, and folie induite. The subtype is determined by the clinical presentation, and the therapy must be customized accordingly [79,106].

Folie imposée was first identified in 1877 by Lasegue and Farlet, and it is supposed to involve a dominant person, known as the ‘primary’, ‘inducer’, or ‘principal’, who initially forms a delusional belief and imposes it on another person, known as the secondary, acceptor, or associate. Folie communiquée was initially referred to by Jules Baillarger in 1860. This represents the contagion of ideas after a long period of initial resistance. Folie induite was described by Lehmann in 1885 and is characterized as a situation in which a delusional individual induces their delusion in an already delusional secondary patient. Folie simultané, which Regis originally mentioned in 1880, refers to the simultaneous beginning of the same hallucination in closely connected persons. Subjects typically exhibit disordered cognitive patterns along with underlying anxiety or despair [107].

In these situations, treatment involves both the patient and family members; separating the secondary instance from the dominant case is the first line of treatment, and antipsychotic medications are necessary for persistent symptoms. The most difficult part can be when family members who have the same delusional beliefs do not approve of any care or separation from the main individual. Therefore, just treating the patients and hoping that their families would see improvements would be the remaining strategy [62,106,108].

By proxy, this is another relatively rare subtype of DP. In these situations, the dominant psychotic person thinks that someone close to them (e.g., the patient’s child or pet, but not the patient themselves) is contaminated with germs. There are very rare situations when both parents project onto their child. The dependents are exposed to the uniquely designed intervention of the dominant psychotic individual even if they may not all have the same hallucinations. DP by proxy falls under the category of Munchausen’s syndrome by proxy, but the parent’s delusions make it worse [109]. In order to prevent any consequences for the child’s physical and mental health, it is mandatory to recognize this condition, as there have been cases of food deprivation and other abusive behaviors secondary to parents’ delusions [110,111,112].

### 7.2. DP and Morgellons Disease

Morgellons disease was first identified in a 2-year-old child when strange filaments described as dandelion fuzz were observed. Usually, in this condition, patients complain about multicolored fibers that emerge from nonhealing wounds, and they describe itching, biting, or crawling sensations in the skin. Some studies suggest that Lyme disease may be connected to Morgellons disease. The Centers for Disease Control and Prevention, however, found no evidence of such a connection with Lyme disease or other well-known infectious diseases. More descriptions of cases appeared after 2002, when data about this assumed disease appeared on the internet, and because of this, it was one of the first illnesses to be socially transmitted over the internet (folie à internet). After many other investigations and considerations, Morgellons disease is considered part of delusional infestation and is not a distinct condition, especially given the fact that even in these patients, their symptoms resolved with antipsychotic treatment [113,114].

### 7.3. DP and Parkinson’s Disease

A few cases in the literature have associated DP with Parkinson’s disease, especially as a secondary side effect of antiparkinsonian treatment. There is no correlation between the duration of the therapy and the start of DP. The general goal of treatment for managing DP and DP psychosis is to limit the use of drugs that are likely to cause psychosis while preventing a major deterioration of motor symptoms [115].

Levodopa or carbidopa can be a viable replacement for a direct agonist since psychotic symptoms are more frequent side effects of these agents. In any case, changes in dopaminergic replacement treatment may aggravate motor symptoms, which may also restrict the ability to cure DP. Antipsychotic treatment can be associated with persistent psychiatric symptoms; low doses of clozapine have been shown to be effective in treating psychotic symptoms in PD without aggravating parkinsonian symptoms [115,116].

Another case that must be taken into consideration is DP as a prodromal symptom of Parkinson’s disease. The reason for this hypothesis is that Parkinson’s disease and DP may involve the same anatomical structure and share some of the same neurotransmitters (somatic delusions and visual and tactile hallucinations may be significantly influenced by the cortico-striatal-thalamus-cortical loop and putamen dysfunction) [117].

### 7.4. DP and Dementia

Delusions and hallucinations can also be experienced by patients in the prodromal stage and early stages of dementia. DP is not specific to patients with neurocognitive disorders and is much less common as a prominent sign of dementia. The estimated prevalence of DP in dementia is approximately 5% [98].

It cannot be determined whether DP symptoms occur preferentially in a particular subtype of dementia. A case of Ekbom syndrome in a patient with multi-infarct dementia was noted. According to a case report, the performed computed tomography scans were able to underline the fact that the damage to the putamen and adjacent brain regions of the somatic/dorsalstriato –thalamus–cortical loop may have had a significant impact on the pathophysiology of DP [118].

Some case reports studied the association between DP and dementia with Lewy bodies (DLB), and it was concluded that patients with symptoms of DP who do not have an established diagnosis of dementia may be considered to have prodromal DLB with psychiatric onset. Regarding the treatment for these cases, an association between acetylcholinesterase inhibitors and antipsychotics can be prescribed. Also, physicians must take into consideration that, due to the strong sensitivity of DLB to antipsychotics, adverse symptoms such as acute-onset parkinsonism, drowsiness, and neuroleptic malignant syndrome can appear. Taking this aspect into account, some patients can be treated only with acetylcholinesterase inhibitors because they have shown promising results in managing cognitive and behavioral symptoms, indicating that they may be a useful first-line treatment for psychotic symptoms in patients with DLB and psychotic symptoms [119,120,121].

### 7.5. DP and Substance Use

Drug usage in DP patients, especially chronic use of amphetamines and cocaine, should be taken into account, and a urine toxicological test must be performed as a routine check-up. Patients must be informed about the connection between illicit drugs and DP and be advised to stop using drugs in order to analyze whether the intensity of their symptoms is reduced without the need for other medication [122,123]. The mechanism that explains the induced psychosis in stimulant usage is the engagement of the mesolimbic dopamine pathway. Another theory available for these cases is that opioid-induced DP is caused by opioid metabolites, which have been proven to block N-methyl-D-aspartate receptors and lead to psychosis. The mu (μ) opioid receptor contributes to pruritus, which can cause feelings of itching and crawling, and all of these lead to delusion [124].

Alcoholic hallucinosis is a symptom of long-term alcohol misuse, and the International Classification of Diseases-11 has categorized it as alcohol-induced psychotic disorder (AIPD). The patient’s vital signs and state of awareness are unaffected, but in AIPD, symptoms such as delusions of persecution, mood swings, and visual, auditory, or tactile hallucinations are present. Patients with AIPD have been known to undergo DP with extended alcohol use [125].

### 7.6. DP and Stroke

Patients who refer to medical services for symptoms of DP should be evaluated for both organic and non-organic sources, as stroke-related delusion of parasitosis cases were reported in some patients. One possible mechanism that can explain psychiatric symptoms is structural damage to neural circuits or disturbance of neurotransmitter systems. Delusions may occur from primary central nervous system injuries when limbic–cortical linkages are disrupted, and an atypical reaction interacts with an unaffected cortex. Before assuming a direct psychiatric cause, neuroimaging should be taken into account for patients who come with DP, especially for older patients or those who have risk factors for stroke [126].

Cases of post-stroke delusion parasitosis were reported, especially for situations of right-side lesions (right temporoparietal cortex), the thalamus, and the putamen (the putamen influences visuotactile perception; through receptive cells, it projects to the parietal cortex, primary somatosensory cortex, and premotor cortex) [127,128].

### 7.7. DP and COVID-19

COVID-19 infection has consequences for various organ systems mainly because of the cytokine storm, which is explained by the proinflammatory immune response within the humoral immune system. Psychiatric symptoms have been described both in the acute phase and after a certain period of the infection. Acute psychotic symptoms after recovery from COVID-19 in patients without a psychiatric history have been described in many case reports. Analyzing all of the data, physicians also consider a distinct postinfectious syndrome and not only an increase in allostatic load due to the pandemic stressors. DP has also been noted in post-COVID-19 infection, and there are two hypotheses that support this: Increases in dopamine in the setting of catecholamine pathway dysregulation have been noted, which has been demonstrated to happen in neuroinflammatory situations and can explain the emergence of psychotic symptoms; secondly, as a direct result of COVID-19, structural damage related to oxidative stress, interstitial edema, or cerebrovascular accidents may also result in neurotransmitter dysregulation and the emergence of a pro-psychotic state [129].

COVID-19 can also determine neurological damage, which is called neuro-COVID. Any cerebral infarction, especially in the basal ganglia (as sequelae of COVID-19), can be associated with the delusions within neuro-COVID, as it has been stated that secondary DP has been highly correlated with anatomical and functional abnormalities in the basal ganglia. Any DP associated with COVID-19 infection can be considered not only as primary DP but also as the second form [130].

The association of DP with non-neurological diseases acknowledges psychosomatic factors, such as stress and anxiety, contributing to the development of delusion. Recognizing the links with underlying medical conditions is crucial, as psychological distress from these conditions can fuel delusions. This underscores the intricate interplay between mental and physical health, emphasizing the need for thorough patient evaluation to exclude other diagnoses. Neglecting proper investigations may result in misdiagnosis and mistreatment in psychiatric settings, risking overlooking serious organic diseases. Addressing both the psychiatric and underlying non-neurological aspects of DP patients ensures a comprehensive treatment plan, averting potential complications [131].

### 7.8. Secondary DP

Clinical cases that report the coexistence of pathologies that might share similar pathophysiological mechanisms describe the two-factor theory. This can be the case of Hakim–Adams syndrome (e.g., normal-pressure hydrocephalus) and Ekbom syndrome described in the same patient. An improved understanding of the physiopathology of DP may result from the relationship between impaired belief evaluation (e.g., frontal cortex) and abnormal cortical processing (e.g., parietal cortex) [132]. Another neurological disease that can hide under the sequelae of DP is posterior reversible encephalopathy syndrome, which is a syndrome of transient vasogenic edema over the white matter of the cerebral posterior regions [133].

The diagnostic challenges regarding DP have been proven in different case reports. One example of such a situation is a case described by the Department of Neurology at Sun Yat-sen University, China, where patients with untreated herpes zoster presented insomnia, mental tension, anxiety caused by itching, and visual hallucinations, secondary to autoimmune system dysregulation, and all of these symptoms can shape the clinical picture of DP. As for the therapy, antipsychotics can be associated with the treatment of the underlying organic condition [134]. Another complication of herpes zoster is postherpetic neuralgia, which can also lead to secondary DP, but very few cases have been reported [32]. Another example of such medical confrontations is the association between Kyrle’s disease and DP. Kyrle’s disease is also a rare dermatological pathology that consists of a disorder of keratinization with follicular and parafollicular hyperkeratotic lesions. From a clinical point of view, a patient can develop superficial lesions secondary to intense pruritus. There is no causal connection between the two diseases, as they can unintentionally develop simultaneously. This case demonstrates how important multidisciplinary collaboration is in treating these patients once again [135].

Another uncommon example of secondary DP was described in patients with severe anemia. One case report described a patient with severe iron deficiency anemia and a fixed delusion of infestation associated with tactile hallucinations. In this case, treating the medical condition resolved all of the psychiatric symptoms [136].

Case reports regarding psychotic disorder due to another medical condition also included DP secondary to HIV infection. The theory that HIV patients have a higher risk of developing psychotic disorders also supports this correlation. As in other cases of secondary DP, antipsychotic treatment has been associated [137].

### 7.9. Drug-Induced DP

Certain drugs are thought to cause DP (e.g., anti-Parkinson medication, antidepressants, antiepileptic agents, prescription stimulants), but no studies have been conducted to establish how pharmacodynamics are linked with these therapies. In this medical context, dopaminergic therapy with ropinirole, amantadine, piribedil, levodopa/carbidopa, pergolide, cabergoline, and pramipexole is considered to be associated with drug-induced DP. Different studies concluded that there might be a correlation between the onset of hallucinations and possible underlying psychiatric conditions; patients who had hallucinations after three months from the beginning of dopaminergic medication had underlying comorbid psychiatric disorders, whereas individuals who developed hallucinations after at least one year of dopaminergic therapy had just primary Parkinson’s disease [138,139,140].

Patients reported hallucinations most commonly described as ‘bugs crawling on my skin’ before developing seizures as a result of a bupropion overdose (a norepinephrine-dopamine reuptake inhibitor). In a very small number of cases, DP was reported after treatment with other antidepressants such as selective serotonin-reuptake inhibitors or tricyclic antidepressants [138,139]. Agents such as lamotrigine, lacosamide, and topiramate can determine tactile hallucinations and further develop in DP [138].

Methylphenidate, atomoxetine, and mixed amphetamine salts used for the treatment of attention deficit hyperactivity disorder were reported to determine DP-associated symptoms in both adults and children [139,141].

Ciprofloxacin and clarithromycin, which are known to determine psychiatric side effects, antihypertensive agents such as propranolol and hydralazine, rifampin for the treatment of tuberculosis, efavirenz as an antiretroviral agent for HIV, and interferon alpha-2B can be associated with DP symptoms [138,139].

## 8. Conclusions and Perspectives

**Figure 2:**
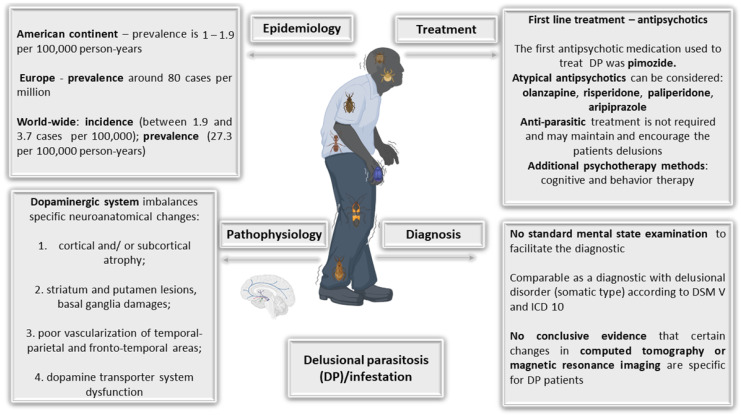
Aspects used for the management of DP.

In the present review, an overview of the management of DP that consolidated the latest available information was presented. Emphasizing early and accurate diagnosis, DP exhibits unique challenges and is influenced by various risk factors. Therapeutic management faces challenges with delayed clinical presentation and the absence of standardized treatments. Case reports, including those associating DP with conditions such as folie à deux and Morgellons disease, provide valuable insights. Figure 2 provides a summary presentation of the most relevant elements in the management of a DP patient.

One of the most significant challenges for physicians in DP cases remains the patient’s conviction of the authenticity of the infestation. Because of this, the patient will receive delayed psychiatric treatment, which will allow a natural evolution of the disease, complicating future compliance.

DP remains a real challenge in medical practice, and most of the time, it involves a multidisciplinary approach. Despite scientific advancements, DP’s management necessitates ongoing research, the development of novel treatments, and personalized approaches to enhance patient outcomes.

## Figures and Tables

**Table 1:** Laboratory tests (not all of them are essential for diagnosis, but they are useful for differential diagnoses) that may be used for DP patients to screen for underlying organic conditions.

Laboratory Parameter	Description in DP Symptomatology
Skin allergy tests	Useful for differential diagnoses and a possible etiology for pruritus
Complete blood count	Focus on increasing eosinophils. Increased eosinophils might be a sign of a parasite or other organism generating symptoms because of the body’s immunological response. It can reveal anemia and hematologic malignancy.
Electrolytes	Electrolyte imbalances (particularly in calcium and phosphorus) may reveal a renal etiology of pruritus
Erythrocyte sedimentation rate	Useful for differential diagnoses if correlated with other laboratory parameters.
C-reactive protein	
Fasting glucose	Secondary DP can be a cause of diabetes mellitus
Lepromin test	Secondary DP can be a cause of infection with leprosy
Liver function	The assessment of liver function can reveal possible underlying hepatobiliary disease and a possible etiology for pruritus
Mantoux tuberculin skin test, interferon-gamma release assays as a blood test	Secondary DP can be a cause of infection with tuberculosis
Serology for *Borrelia burgdorferi*	Secondary DP can be a cause of Lyme disease
Serology for Hepatitis B and C	Secondary DP can be a cause of infection with hepatitis
Serology for HIV infection	Secondary DP can be a cause of infection with HIV virus
Serology for *Treponema pallidum*	Secondary DP can be a cause of infection with syphilis
Serum creatinine	Abnormal levels of creatinine may reveal a renal etiology of pruritus
Stool specimens	Useful for differential diagnosis with gastrointestinal parasitic infections
Thyroid-stimulating hormone, levels of the circulating thyroid hormones triiodothyronine (T3) and thyroxine (T4)	An evaluation for thyroid function and possible disorders, such as hyperthyroidism or hypothyroidism
Urine analysis	Secondary DP can be a cause of substance abuse, including methamphetamine use, alcohol withdrawal, and acute cocaine use (described as “cocaine bugs”)
Vitamin B and folate levels	Secondary DP can be a cause of medical illnesses such as B12 and folate deficiencies

**Table 2:** A brief characterization of antipsychotics with potential use in DP.

Molecule	Daily Dose (mg)	Outcomes	Side Effects	Ref
Generation I (typical)				
Chlorpromazine	150–300	Partial remission	Cholestasis and pro-inflammatory responses	[92,93]
Pimozide	1–12	Full remission for 48% of the patients and partial remission for 52%	Life-threatening arrhythmias	[93,94]
Haloperidol	1–10	Full remission for 66.6% of the patients and partial remission for 33.3%	Extrapyramidal reactions	[93,95]
Trifluoperazine	2–15	Full remission for 43% of the patients, partial remission for 43% of the patients, no effect for 14% of the patients		[93,96]
Generation II (atypical)				
Amisulpride	200–400	Partial remission	Enhancement of the prolactin level	[58,93]
Risperidone	0.5–3	Full remission for daily doses between 0.5–1 mg and partial remission for 1–3 mg	Sedation and enhancement of the prolactin level	
Quetiapine	50–300	Partial remission	Sedation, enhancement of the prolactin level, weight gain	
Olanzapine	2.5–10		Sedation, significant weight gain, cardiovascular risk	
